# Association of Alcohol Drinking Patterns with Metabolic Syndrome and Its Components in Korean Adults: The Korea National Health and Nutrition Examination Survey 2016–2018

**DOI:** 10.3390/ijerph18126433

**Published:** 2021-06-14

**Authors:** Seung Won Lee, Sung-In Jang

**Affiliations:** 1 Institute of Pharmaceutical Science, Korea University, Sejong 30019, Korea; lseungwon84@gmail.com; 2 Department of Preventive Medicine, Yonsei University College of Medicine, Seoul 03722, Korea

**Keywords:** alcohol consumption, alcohol drinking patterns, metabolic syndrome, epidemiology

## Abstract

This study examined the association of alcohol drinking patterns with metabolic syndrome (MetS) and its components in a nationally representative sample of South Korean adults. The cross-sectional study included 12,830 current drinkers (6438 men and 6392 women) who were at least 20 years old. Measures of alcohol drinking patterns included average drinking frequency, usual quantity, and binge drinking frequency over the past year. Multivariate logistic regression was performed to estimate odds ratios and 95% confidence intervals for MetS and its components according to alcohol drinking patterns, and also to examine linear trends in these relationships. The prevalence of MetS was 1822 (26.2%) in men and 1313 (17.5%) in women. After adjusting for potential confounding factors, drinking quantity and binge drinking frequency were positively associated with MetS in both sexes. Regarding components of MetS, while the risk of low HDL cholesterol decreased as drinking frequency increased, other MetS components (abdominal obesity, high blood pressure, and impaired fasting glucose) worsened. Our results suggest that separate management of each component of MetS will be required to protect cardio-metabolic health, and a healthy drinking culture that refrains from binge drinking should be established in the context of public health.

## 1. Introduction

The metabolic syndrome (MetS) is a cluster of risk factors defined by abdominal obesity, high blood pressure, elevated fasting glucose, and dyslipidemia, and is well known to be associated with type 2 diabetes, cardiovascular disease, and mortality [1,2,3]. The prevalence of MetS is estimated to be approximately 20–25% in adults worldwide [4]. According to Korean National Health Insurance Service data from 2009 to 2013, the age-adjusted prevalence of MetS increased from 28.84 to 30.52% among adults over the age of 30 in the Republic of Korea [5]. MetS poses a major challenge for public health professionals and policy makers. Because living habits such as alcohol drinking, smoking, and physical activity are known to be major risk factors for MetS and its components, recommendations for a healthy lifestyle are needed [6,7].

Regarding alcohol consumption, one of the most common health-related behaviors, it has been reported that a small amount of alcohol has a positive effect on health, but excessive drinking results in serious health problems, including an increased risk of cardiovascular disease, cancer, liver cirrhosis, chronic pancreatitis, and violence [8,9,10].

The association between MetS and alcohol consumption is complex, with previous studies reporting both protective and detrimental effects [11,12,13,14,15,16]. The associations between alcohol consumption and cardiovascular outcomes also remain controversial [17,18]. The controversy could be related to the complex mechanistic relationships between alcohol consumption and each component of MetS. Previous studies have reported that alcohol consumption has a U-shaped, J-shaped, or positive relationship with blood pressure [19,20,21,22], glucose metabolism [23,24], and triglyceride concentration [25,26,27], whereas the relationship with high-density lipoprotein (HDL) cholesterol was negative [28,29,30]. Given these results, in order to understand the relationship between alcohol consumption and MetS as a risk factor for cardiovascular disease, it is necessary to consider the associations between alcohol consumption and each of the various components of MetS. In addition, the inconsistent results in these reports could be attributable in part to variations in alcohol drinking patterns [20,30]. However, only limited evidence has been reported on associations between components of MetS and alcohol drinking patterns, including the frequency of drinking, usual quantity, and binge drinking frequency.

The average per capita alcohol consumption in the Republic of Korea is 10.2 L (as pure alcohol), higher than the World Health Organization Western Pacific average of 7.3 L [31]. Additionally, 13.9% of Koreans were found to suffer from alcohol use disorders, and 5.5% from alcohol dependence, well above the WHO Western Pacific Regional averages of 4.7% and 2.3%, respectively [31]. In the context of the population studied herein, guidelines for proper drinking patterns may be expected to decrease the risk of alcohol-related diseases and to improve health at the national level. Thus, this study investigated the associations between alcohol drinking patterns with MetS and its components in a sample representing South Korean adults in order to help give some guidelines on drinking habits to the general population at the national level.

## 2. Materials and Methods

### 2.1. Study Population

This study used data from the seventh Korean National Health and Nutrition Examination Survey 2016–2018 (KNHANES VII), a nationally representative survey conducted by the Korean Ministry of Health and Welfare in South Korea [32]. Each year from 2016 to 2018, 192 districts and 4416 households were randomly sampled nationwide. A total of 24,269 participants selected from a total of 10,611 households had health examinations and questionnaire surveys about health-related behaviors, including alcohol drinking patterns. The present study examined data for 13,240 current alcohol drinkers aged 19 years and older, and after excluding those for whom information about components of MetS was missing, and measurement outliers, 12,830 people were finally included in the present study.

The KNHANES received approval from the Institutional Review Board of the Korea Centers for Disease Control and Prevention (KCDC). All participants in the survey provided written informed consent.

### 2.2. Measurements

#### 2.2.1. MetS and Its Components

Our definitions of MetS and its components were based on the National Cholesterol Education Program Adult Treatment Panel III [33] and the Korean Society for the Study of Obesity [34]. MetS was defined as the presence of three or more of the following criteria: (1) abdominal obesity (waist circumference ≥90 cm in men and ≥85 cm in women), (2) high blood pressure (systolic blood pressure ≥130 mmHg or diastolic blood pressure ≥85 mmHg) or taking antihypertensive medication, (3) impaired fasting glucose ≥100 mg/dL or being treated for diabetes, (4) hypertriglyceridemia (≥150 mg/dL), (5) low HDL cholesterol (≤40 mg/dL in men and ≤50 mg/dL in women).

Waist circumference was measured for each participant at a level midway between the lowest lateral border of the ribs and the uppermost lateral iliac crest, with the participant standing. Body weight and height were measured in light indoor clothing, without shoes, to the nearest 0.1 kg and 0.1 cm, respectively. Blood pressure was measured in the right arm using a standard mercury sphygmomanometer (Baumanometer, Ellicott City, MD, USA). Blood pressure was measured three times, and the average of the second and third measurements was used. Venous blood samples were collected from subjects after they had fasted for at least 8 h. Levels of total cholesterol, triglycerides, HDL, and fasting blood glucose were measured by enzymatic methods using a model 7600–210 Automatic Analyzer (Hitachi, Tokyo, Japan) in a certified clinical laboratory.

#### 2.2.2. Alcohol Drinking Patterns

Self-reported questionnaires were used to assess each participant’s alcohol drinking patterns, including average drinking frequency, usual quantity per drinking day, and the frequency of binge drinking during the last year. Average drinking frequency in the past year was classified as less than once a month, once a month, 2–4 times a month, 2–3 times a week, or more than 4 times a week. Usual drinking quantity per drinking day was classified as 1–2 drinks, 3–4 drinks, 5–6 drinks, 7–9 drinks, or 10 or more drinks. The frequency of binge drinking was classified as none, less than once a month, once a month, once a week, or every day. One drink was defined as 30 mL of liquor or 200 mL of beer, equivalent to 10 g of pure alcohol. Binge drinking was defined as the consumption on a single occasion of 7 or more drinks in men, and 5 or more drinks in women.

#### 2.2.3. Statistical Analyses

Data for men and women were analyzed separately. Gender differences were analyzed using the independent *t*-test for continuous variables and the chi-squared test for categorical variables. Odds ratios (OR) and 95% confidence intervals (95% CI) for MetS were calculated after adjusting for confounding variables (age, education level, household income, current smoking, regular physical activity, region, occupation, year, and menopausal status for women). Associations of MetS and its components according to alcohol drinking patterns were investigated using a multivariate logistic regression analysis. To detect linear trends in these relationships, multivariate linear regression was also used. Smoking status was categorized as currently smoking or currently nonsmoking; the nonsmoking group included ex-smokers and non-smokers. Household income was divided into quartiles (low, medium-low, medium-high, and high income). The educational level was classified as below middle school or above middle school. Regular exercise was defined as at least one of the following conditions: aerobic physical activities of vigorous intensity lasting more than 20 min and performed 3 days each week, or aerobic physical activities of moderate intensity lasting more than 30 min performed 5 days each week, or walking for more than 30 min performed 5 days each week. Occupations were classified as white collar, sales and services, blue collar, or unemployed. Region was defined as urban or rural. All analyses were performed using SAS statistical version 9.4 (SAS Institute Inc., Cary, NC, USA). All statistical tests were two-sided, and values of *p* less than 0.05 were considered statistically significant.

## 3. Results

The general characteristics of the study population are presented in Table 1. This study included 6116 men (49.8%) with a mean age of 44.5 years and 6157 women (50.2%) with a mean age of 43.9 years. Mean waist circumference, BMI, blood pressure, fasting glucose, and triglyceride levels were significantly higher in men than in women. However, mean total cholesterol and HDL cholesterol were significantly higher in women than in men. Regarding alcohol drinking patterns, the drinking frequency reported by the highest proportion of men was 2–4 times/month (32.5%). Conversely, the drinking frequency reported by the highest proportion of women was less than once a month (33.8%). The drinking quantity reported by the highest proportion of men was 7–9 drinks at a time (22.7%), but was 1–2 drinks for women (49.7%). The frequency of binge drinking reported by the highest proportion of men was once a week (26.9%), but the highest proportion of women reported none (53.1%). Incidences of MetS were 1729 (26.1%) and 1265 (17.4%) in men and women, respectively.

Table 2 shows the OR and 95% CI for MetS, according to alcohol drinking patterns, among people who had consumed alcohol in the past year. When adjusted for potential confounding factors, drinking frequency was not significantly associated with MetS in men. The odds ratios for MetS among women who consumed alcohol 2–4 times/month (OR 0.76, 95% CI 0.61–0.95) or 2–3 times/week (OR 0.65, 95% CI 0.49–0.85) were lower than for those who consumed alcohol less than once a month. A significant linear trend between drinking frequency and MetS was observed in both men and women (*p* for linear trend = 0.0128 in men and 0.0088 in women). Linear trends for the association between usual drinking quantity and MetS were also significant (*p* < 0.0001 in men, and <0.0001 in women). Men who consumed 7–9 drinks or ≥10 drinks per drinking day had greater risk of MetS than those who consumed 1–2 drinks per drinking day, with the highest risk being among those who consumed ≥ 10 drinks per drinking day (OR 1.89, 95% CI 1.48–2.41). In women, those who consumed 5–6 drinks, 7–9 drinks, or ≥10 drinks per drinking day had increased risk of MetS, and this risk was highest in those who consumed ≥10 drinks per drinking day (OR 2.26, 95% CI 1.49–3.43). The linear relationship between binge drinking frequency and MetS was also significant in men (*p* < 0.0001), but its statistical significance was borderline in women (*p* = 0.0631). Compared to the reference group (no binge drinking), the odds ratios for MetS were significantly higher in men who reported binge drinking once a week (OR 1.64, 95% CI 1.35–2.00) or every day (OR 1.84, 95% CI 1.47–2.31). In women, odds ratios for MetS were significantly higher in those who reported binge drinking once a month (OR 1.37, 95% CI 1.02–1.84) or once a week (OR 1.38, 95% CI 1.00–1.89) than in women who reported no binge drinking.

Table 3 and Table 4 present the OR and 95% CI for each component of MetS according to alcohol drinking patterns in men and women. In men (Table 3), drinking frequency had a significant linear trend with high blood pressure (*p* < 0.0001), impaired fasting glucose (*p* = 0.0004), hypertriglyceridemia (*p* < 0.0001), and low HDL cholesterol (*p* < 0.0001). The risks of high blood pressure and hypertriglyceridemia were significantly higher in men who consumed alcohol 2–3 times/week (OR 1.47, 95% CI 1.19–1.83 for high blood pressure; OR 1.29, 95% CI 1.05–1.58 for hypertriglyceridemia) or ≥4 times/week (OR 1.96, 95% CI 1.51–2.54 for high blood pressure; OR 1.40, 95% CI 1.10–1.79 for hypertriglyceridemia) than those who consumed alcohol less than once a month, after adjusting for potential confounding factors. The risk of impaired fasting glucose was significantly higher in men who consumed alcohol ≥ 4 times/week (OR 1.33, 95% CI 1.02–1.79) than those who consumed alcohol less than once a month. However, the risk of low HDL cholesterol was significantly lower in men who consumed alcohol 2–3 times/week (OR 0.70, 95% CI 0.55–0.90) or ≥4 times/week (OR 0.45, 95% CI 0.34–0.61) than those who consumed alcohol less than once a month. In addition, men who consumed alcohol 2–4 times/month had lower risk of abdominal obesity (OR 1.75, 95% CI 0.58–0.98) than those who consumed alcohol less than once a month.

In women (Table 4), drinking frequency had a linear relationship with abdominal obesity (*p* for linear trend = 0.0448), high blood pressure (*p* = 0.0155), and low HDL cholesterol (*p* < 0.0001). Women who consumed alcohol 2–4 times/month had lower risks of abdominal obesity (OR 0.76, 95% CI 0.62–0.93), hypertriglyceridemia (OR 0.79, 95% CI 0.64–0.98), and low HDL cholesterol (OR 0.70, 95% CI 0.60–0.82) than those who consumed alcohol less than once a month. In addition, the risk of low HDL cholesterol was significantly lower in all groups that consumed alcohol one or more times/month compared to those who consumed alcohol less than once a month. In both men and women, the usual quantity per drinking day had linear associations with abdominal obesity (*p* for linear trend < 0.0001 in men, *p* < 0.0001 in women), high blood pressure (*p* < 0.0001 in men, *p* < 0.0001 in women), impaired fasting glucose (*p* < 0.0001 in men, *p* < 0.0001 in women), and hypertriglyceridemia (*p* < 0.0001 in men, *p* = 0.0125 in women). The risks of abdominal obesity and high blood pressure were significantly higher in men who usually consumed 7 or more drinks than in men whose usual quantity was 1–2 drinks. The risks of impaired fasting glucose and hypertriglyceridemia were significantly higher in men who usually consumed 5 or more drinks. On the other hand, men who consumed 5–6 drinks had lower risk of low HDL cholesterol (OR 0.76, 95% CI 0.60–0.96). In women, the risk of abdominal obesity was higher among those whose usual consumption was 3 or more drinks than in those who consumed 1–2 drinks. The risk of high blood pressure was higher in women who usually consumed 5–6 drinks (OR 1.89, 95% CI 1.43–2.50) or 7–9 drinks (OR 2.07, 95% CI 1.51–2.85). The risk of impaired fasting glucose was higher in women who consumed 7–9 drinks (OR 1.52, 95% CI 1.13–2.06) or ≥10 drinks (OR 1.70, 95% CI 1.15–2.53). The risk of hypertriglyceridemia was higher in women who consumed 5–6 drinks (OR 1.41, 95% CI 1.08–1.86) or ≥10 drinks (OR 1.77, 95% CI 1.17–2.67) compared to those who usually consumed 1–2 drinks. The frequency of binge drinking had linear associations with abdominal obesity (*p* for linear trend = 0.0154 in men, *p* < 0.0001 in women), high blood pressure (*p* < 0.0001 in both sexes), impaired fasting glucose (*p* < 0.0001 in men, *p* = 0.0059 in women), hypertriglyceridemia (*p* < 0.0001 in men), and low HDL cholesterol (*p* < 0.0001 in both sexes). Men who reported binge drinking once a month had higher risk of hypertriglyceridemia (OR 1.23, 95% CI 1.02–1.49) than those who reported none. The risks of abdominal obesity, high blood pressure, impaired fasting glucose, and hypertriglyceridemia were also higher in men who reported binge drinking once a week or every day compared to those who reported none. On the other hand, the risk of low HDL cholesterol was significantly lower among men who reported binge drinking once a week or every day than those who reported none. The risk of abdominal obesity was significantly higher in women who reported binge drinking less than once a month (OR 1.24, 95% CI 1.01–1.52) than in those who reported none. The risk of high blood pressure was significantly higher in all groups who reported binge drinking one or more times a month, but the risk of low HDL cholesterol was significantly lower than in those who did not binge drink. In addition, women who reported binge drinking every day had higher risks of impaired fasting glucose (OR 1.97, 95% CI 1.26–3.09) and hypertriglyceridemia (OR 1.76, 95% CI 1.08–2.87).

## 4. Discussion

We investigated the association of alcohol drinking patterns with MetS and its components among Korean adults. Drinking frequency was significantly associated with MetS only in women. Adjusting for potential confounders, women who consumed alcohol at least 2–4 times/month had lower risk of MetS than women who consumed alcohol less than once a month. This seems to be due to the relatively large influence of inverse associations between drinking frequency and HDL cholesterol and triglyceride levels. In addition, there was no significant association in women between drinking frequency and impaired fasting glucose.

A cross-sectional study analyzing data from the 1999–2002 National Health and Nutrition Examination Survey (NHANES) reported that people who consumed alcohol 1–2 times/week showed significantly lower risk of MetS than those who drank less than once a week, after adjusting for age; sex; race; years of education; family history of coronary heart disease, stroke, or diabetes; dietary practice (saturated fat consumption, dietary fiber consumption); cigarette smoking status; physical activity; usual quantity of drinking; and frequency of binge drinking [20]. In addition, when analyzed according to each component of MetS, drinking frequency had a significant inverse association with low HDL cholesterol and a positive relationship with blood pressure. In accordance with that previous study, our study found drinking frequency to be positively associated with high blood pressure and inversely associated with low HDL cholesterol. Another previous study analyzing data from KNHANES in 2008 reported that drinking frequency was not associated with MetS, either in men or in women [28]. However, when analyzed by each component of MetS, men who usually consumed alcohol 4 or more times/week had a lower OR for low HDL cholesterol than those who usually consumed alcohol less than once a month, and women who usually consumed alcohol 2–3 times/week had lower OR for low HDL cholesterol and higher OR for high blood pressure than those who usually consumed alcohol less than once a month. Another Korean study reported men who usually consumed alcohol 2–3 times/week or more had a lower risk of low HDL cholesterol, and a higher risk of high blood pressure and impaired fasting glucose, than non-drinking men [29]. Women who usually consumed alcohol 2–3 times/week or more had a significantly lower risk of low HDL cholesterol than non-drinkers. A cross-sectional study among the general Japanese population reported a positive association between drinking frequency and high blood pressure, and an inverse association with dyslipidemia, where dyslipidemia was defined as a triglyceride level >150 mg/dL, a low-density lipoprotein cholesterol level >140 mg/dL, or an HDL cholesterol level <40 mg/dL [30]. Summarizing the results of previous studies, most found that blood pressure had a positive relationship with alcohol drinking frequency and HDL cholesterol levels had a negative relationship, which differed by sex [20,28,30,35]. As in the previous studies, our study found that men who consumed alcohol 2–3 times/week or more had significantly higher blood pressure than men who consumed alcohol less than once a month, whereas the risk of low HDL cholesterol was reduced with increased drinking frequency in a linear relationship that was statistically significant. Men and women who consumed alcohol 2–4 times/month had lower risks of abdominal obesity, hypertriglyceridemia (only in women), and low HDL cholesterol.

Regarding the usual drinking quantity, previous studies reported that men who consumed at least 5–6 drinks per drinking day had higher risks of abdominal obesity, high blood pressure, impaired fasting glucose, and hypertriglyceridemia [28,35]. Some studies also reported that men who consumed 3–4 drinks per drinking day had increased risks of abdominal obesity, high blood pressure, impaired fasting glucose, hypertriglyceridemia, and MetS [20,28]. Previous studies also found that women who consumed at least 3–4 drinks per drinking day had increased risks of abdominal obesity, high blood pressure, impaired fasting glucose, and hypertriglyceridemia relative to those who consumed 1–2 drinks per drinking day [20,28,35]. In our study, women who consumed at least 3–4 drinks per drinking day had increased risk of abdominal obesity. In addition, abdominal obesity, high blood pressure, impaired fasting glucose, and hypertriglyceridemia all had linear relationships with usual drinking quantity in both sexes.

Previous studies that investigated the frequency of binge drinking found that, even if binge drinking occurred less than once a week, women had higher risks of abdominal obesity and hypertriglyceridemia, relative to those who did not binge drink, and those who reported binge drinking more often had increased risks of other MetS components, with the exception of low HDL cholesterol [20,28,35]. Our study also observed that women who engaged in binge drinking less than once a week had an increased risk of abdominal obesity, and those who reported binge drinking more often had increased risks of other MetS components, with the exception of low HDL cholesterol. Men who reported binge drinking once a week or more had increased risks of all components of MetS, relative to those who did not binge drink at all, again with the exception of low HDL cholesterol.

Binge drinking has also been reported in the general population to deteriorate neurological function and increase the risk of dementia and cerebrovascular disease, several types of cancer including the oral cavity, pharynx, esophagus, liver, colon, rectum, and breast (in women) [36,37]. Cardiovascular disease, cerebrovascular disease, dementia, and cancer, which can increase the risk of incidence due to binge drinking, are major causes of death in Korea as well as in the world [38]. Therefore, in order to reduce the burden of disease and the mortality rate, national efforts to create such a social environment will be needed, along with individual efforts to refrain from binge drinking.

Some previous studies have suggested that light or moderate alcohol drinking plays a protective role in cardio-metabolic health [11,39,40]. Our study showed that women who drank alcohol 2–4 times/month or more had lower OR for MetS than those who consumed alcohol less than once a month. The result may be largely attributable to the effect of alcohol drinking on HDL cholesterol level. Previous studies reported the cardio-metabolic protective effect of alcohol consumption to be based on the relationship between elevation of HDL cholesterol and alcohol consumption. However, it should not be overlooked that the elevation of HDL cholesterol by alcohol consumption may occur together with increased risk of other components of MetS [13,20,28]. Previous studies have found that the association between low HDL cholesterol and drinking frequency is opposite to that of high blood pressure [11,28,30]. Our study also found that women who drank alcohol 4 times/week or more had a lower risk of low HDL cholesterol than those who drank alcohol less than once a month, but a greater risk of high blood pressure. In addition, studies in Russia reported that Russians had higher HDL levels than Western Europeans and Americans, but they also had higher age-adjusted rates of cardiovascular disease and all-cause mortality [41,42]. Therefore, the favorable effects of drinking alcohol on HDL cholesterol levels should not, in itself, be interpreted as conferring a protective effect on overall cardio-metabolic health.

In this regard, because associations between the components of MetS and drinking patterns have been shown to vary according to sex and ethnicity, it is necessary to investigate how each component of MetS is related to drinking patterns when we investigate the effect of drinking patterns on cardio-metabolic health. For example, studies examining the mechanism of impaired fasting glucose, one of the components of MetS, indicate that it is linked to insulin resistance, and some studies have reported conflicting directions of the relationship between alcohol consumption and insulin resistance [43,44]. Moreover, we observed that, even if the level of one component (HDL cholesterol) improved as drinking frequency increased or as the usual amount of alcohol consumption increased, another component of MetS worsened. Therefore, the inverse relationship between drinking frequency and MetS observed in our study needs to be carefully evaluated in terms of each component of MetS.

This study has some limitations. First, given the cross-sectional design of our study, it was impossible to derive causal inferences about associations between drinking patterns and components of MetS. Second, we used self-reported data on drinking patterns through questionnaire, and thus recall bias and miscategorization of drinking habits are possible. Third, other aspects of drinking behavior, such as drinking with meals and preferred types of alcoholic beverages, were not included in the analysis. In particular, different types of alcohol may be composed of different substances and contribute to different effects, which may affect study findings. However, there are also distinct advantages to the data analyzed here. First, our study population was randomly sampled and is representative of the Korean population. Second, various potential confounding variables that can affect the relationship between drinking patterns and MetS components were considered, and we included statistical tests to evaluate linear relationships.

## 5. Conclusions

The current study observed that drinking patterns have linear relationships with MetS and its components. Drinking quantity and binge drinking frequency were positively associated with MetS. On the other hand, drinking frequency was inversely associated with MetS in women, which may be attributable to its favorable effect on HDL cholesterol level. Although the risk of low HDL cholesterol decreased as drinking frequency increased, other MetS components changed adversely, so that frequent drinking cannot be said to prevent or relieve the risk of cardiovascular disease. Our results suggest that separate management of each component of MetS will be required to preserve cardio-metabolic health and that a healthy drinking culture that refrains from binge drinking should be established in the context of public health.

## Figures and Tables

**Table 1:** General characteristics of the study population.

Variables	Total (*n* = 12,273)	Men (*n* = 6116)	Women (*n* = 6157)	*p*-Value
Age (year)	44.2 ± 0.2	44.5 ± 0.3	43.9 ± 0.3	<0.0001
Waist circumference (cm)	82.2 ± 0.1	86.2 ± 0.1	77.2 ± 0.2	<0.0001
BMI (kg/m^2^)	23.9 ± 0.0	24.6 ± 0.1	23.1 ± 0.1	<0.0001
SBP (mm Hg)	116.7 ± 0.2	119.9 ± 0.2	112.7 ± 0.3	<0.0001
DBP (mm Hg)	76.4 ± 0.1	78.8 ± 0.2	73.4 ± 0.2	<0.0001
Fasting glucose (mg/dL)	99.1 ± 0.2	102.0 ± 0.4	95.6 ± 0.3	<0.0001
Total cholesterol (mg/dL)	193.0 ± 0.4	192.8 ± 0.6	193.3 ± 0.5	0.0008
HDL cholesterol (mg/dL)	51.7 ± 0.2	47.8 ± 0.2	56.5 ± 0.2	<0.0001
Triglyceride (mg/dL)	139.9 ± 1.4	166.0 ± 2.2	107.6 ± 1.3	<0.0001
Education level				<0.0001
Below middle school	1736 (9.7)	714 (7.5)	1022 (12.4)	
Above middle school	10,537 (90.3)	5402 (92.5)	5135 (87.6)	
Household income				0.1742
Low	1723 (12.1)	851 (11.5)	872 (12.9)	
Medium-low	2911 (22.8)	1409 (22.1)	1502 (23.7)	
Medium-high	3599 (30.7)	1793 (31.2)	1806 (30.1)	
High	4040 (34.3)	2063 (35.2)	1977 (33.3)	
Region				0.0027
Urban	10,282 (87.0)	5062 (86.4)	5220 (87.7)	
Rural	1991 (13.0)	1054 (13.6)	937 (12.3)	
Occupation				<0.0001
White collar	3625 (32.2)	1956 (35.0)	1669 (28.7)	
Sales and services	1768 (15.1)	681 (12.4)	1087 (18.3)	
Blue collar	1906 (15.8)	1564 (24.7)	342 (4.8)	
Unemployed	4974 (36.9)	1915 (27.9)	3059 (48.1)	
Regular exercise	5712 (49.4)	2969 (51.5)	2743 (46.8)	<0.0001
Current smoking	2721 (25.3)	2307 (39.7)	414 (7.4)	<0.0001
Drinking frequency				<0.0001
Less than once a month	3119 (23.5)	899 (15.3)	2220 (33.8)	
Once a month	1590 (12.9)	651 (10.9)	939 (15.5)	
2–4 times a month	3796 (32.3)	1918 (32.5)	1878 (32.1)	
2–3 times a week	2595 (22.1)	1718 (28.1)	877 (14.7)	
More than 4 times a month	1173 (9.1)	930 (13.2)	243 (4.0)	
Usual drinking quantity				<0.0001
1–2 drinks	4600 (32.5)	1271 (18.5)	3329 (49.7)	
3–4 drinks	2608 (20.6)	1220 (18.6)	1388 (23.0)	
5–6 drinks	1761 (15.5)	1109 (18.5)	652 (11.7)	
7–9 drinks	1792 (16.6)	1330 (22.7)	462 (9.0)	
10 or more drinks	1512 (14.9)	1186 (21.7)	326 (6.5)	
Binge drinking frequency				<0.0001
None	4941 (34.7)	1388 (19.8)	3553 (53.1)	
Less than once a month	2417 (20.9)	1201 (20.5)	1216 (21.3)	
Once a month	1919 (17.7)	1177 (20.7)	742 (14.0)	
Once a week	2123 (19.0)	1607 (26.9)	516 (9.1)	
Every day	873 (7.8)	743 (12.1)	130 (2.4)	
Menopause	-	-	2459 (33.1)	
Metabolic syndrome	2994 (22.2)	1729 (26.1)	1265 (18.1)	<0.0001

Values are presented as mean ± standard error or as number (%). Values of *p* compare men and women using independent *t*-test, the Wilcoxon rank sum test, or chi-squared test. Abbreviations: BMI, body mass index; DBP, diastolic blood pressure; HDL, high-density lipoprotein; SBP, systolic blood pressure.

**Table 2:** Associations between alcohol drinking patterns and metabolic syndrome.

Variables	Men		Women	
	OR	95% CI	OR	95% CI
Drinking frequency ^a^				
Less than once a month	1.00		1.00	
Once a month	0.94	(0.71–1.25)	0.88	(0.68–1.13)
2–4 times a month	0.90	(0.71–1.15)	0.76	(0.61–0.95)
2–3 times a week	1.17	(0.92–1.49)	0.65	(0.49–0.85)
More than 4 times a week	1.19	(0.90–1.56)	0.66	(0.43–1.03)
*p* for linear trend	0.0128		0.0088	
Usual drinking quantity ^b^				
1–2 drinks	1.00		1.00	
3–4 drinks	1.04	(0.83–1.29)	1.14	(0.92–1.41)
5–6 drinks	1.23	(0.97–1.55)	1.61	(1.20–2.16)
7–9 drinks	1.64	(1.30–2.07)	2.02	(1.44–2.84)
10 or more drinks	1.89	(1.48–2.41)	2.26	(1.49–3.43)
*p* for linear trend	<0.0001		<0.0001	
Binge drinking frequency ^c^				
None	1.00		1.00	
Less than once a month	1.01	(0.82–1.25)	1.00	(0.79–1.26)
Once a month	1.18	(0.94–1.48)	1.37	(1.02–1.84)
Once a week	1.64	(1.35–2.00)	1.38	(1.00–1.89)
Every day	1.84	(1.47–2.31)	1.36	(0.78–2.38)
*p* for linear trend	<0.0001		0.0631	

^a^ Adjusted for age, education level, household income, occupation, residence area, current smoking, regular physical activity, menopausal status (for women), year, and usual drinking quantity. ^b^ Adjusted for age, education level, household income, occupation, residence area, current smoking, regular physical activity, menopausal status (for women), year, and drinking frequency. ^c^ Adjusted for age, education level, household income, occupation, residence area, current smoking, regular physical activity, menopausal status (for women), and year. Abbreviations: CI, confidence interval; OR, odds ratio.

**Table 3:** The association between alcohol drinking patterns and components of metabolic syndrome in men.

Variables	Abdominal Obesity			High Blood Pressure			Impaired Fasting Glucose			Hypertriglyceridemia			Low HDL Cholesterol		
	N (%)	OR	95% CI	N (%)	OR	95% CI	N (%)	OR	95% CI	N (%)	OR	95% CI	N (%)	OR	95% CI
Drinking frequency ^a^															
Less than once a month	130 (14.5)	1.00		355 (39.5)	1.00		321 (35.7)	1.00		310 (34.5)	1.00		253 (28.1)	1.00	
Once a month	102 (15.7)	0.83	(0.60–1.15)	263 (40.4)	0.99	(0.76–1.29)	249 (38.3)	1.06	(0.81–1.39)	229 (35.2)	1.03	(0.80–1.32)	199 (30.6)	1.07	(0.83–1.39)
2–4 times a month	285 (14.9)	0.75	(0.58–0.98)	776 (40.5)	0.91	(0.73–1.14)	725 (37.8)	0.89	(0.71–1.11)	714 (37.2)	0.91	(0.74–1.11)	505 (26.3)	1.00	(0.80–1.25)
2–3 times a week	293 (17.1)	0.81	(0.61–1.07)	928 (54.0)	1.47	(1.19–1.83)	828 (48.2)	1.21	(0.96–1.52)	790 (46.0)	1.29	(1.05–1.58)	364 (21.2)	0.70	(0.55–0.90)
More than 4 times a week	158 (17.0)	0.74	(0.54–1.02)	629 (67.6)	1.96	(1.51–2.54)	539 (58.0)	1.33	(1.02–1.74)	456 (49.0)	1.40	(1.10–1.79)	163(17.5)	0.45	(0.34–0.61)
*p* for linear trend		0.3177			<0.0001			0.0004			<0.0001			<0.0001	
Usual drinking quantity ^b^															
1–2 drinks	161 (12.7)	1.00		576 (45.3)	1.00	-	513 (40.4)	1.00		412 (32.4)	1.00		361 (28.4)	1.00	
3–4 drinks	166 (13.6)	1.10	(0.85–1.42)	604 (49.5)	1.04	(0.86–1.26)	539 (44.2)	1.05	(0.85–1.30)	438 (35.9)	1.07	(0.88–1.30)	305 (25.0)	0.80	(0.64–1.00)
5–6 drinks	160 (14.4)	1.24	(0.92–1.68)	517 (46.6)	1.18	(0.95–1.46)	484 (43.6)	1.33	(1.05–1.67)	450 (40.6)	1.24	(1.00–1.54)	256 (23.1)	0.76	(0.60–0.96)
7–9 drinks	218 (16.4)	1.49	(1.13–1.96)	704 (52.9)	1.48	(1.20–1.83)	636 (47.8)	1.68	(1.34–2.10)	596 (44.8)	1.40	(1.14–1.71)	297 (22.3)	0.79	(0.61–1.02)
10 or more drinks	263 (22.2)	2.18	(1.62–2.93)	550 (46.4)	1.56	(1.24–1.96)	490 (41.3)	1.70	(1.33–2.16)	603 (50.8)	1.76	(1.41–2.18)	265 (22.3)	0.82	(0.63–1.07)
*p* for linear trend		<0.0001			<0.0001			<0.0001			<0.0001			0.1744	
Binge drinking frequency ^c^															
None	194 (14.0)	1.00		671 (48.3)	1.00		607 (43.7)	1.00		447 (32.2)	1.00		403 (29.0)	1.00	
Less than once a month	175 (14.6)	1.07	(0.83–1.38)	487 (40.6)	1.01	(0.84–1.21)	434 (36.1)	1.05	(0.85–1.30)	423 (35.2)	1.14	(0.94–1.39)	311 (25.9)	0.91	(0.75–1.10)
Once a month	161 (13.7)	1.01	(0.76–1.35)	477 (40.5)	1.10	(0.90–1.36)	447 (38.0)	1.16	(0.93–1.44)	460 (39.1)	1.23	(1.02–1.49)	321 (27.3)	0.92	(0.75–1.12)
Once a week	299 (18.6)	1.38	(1.10–1.73)	836 (52.0)	1.70	(1.41–2.06)	770 (47.9)	1.68	(1.37–2.06)	773 (48.1)	1.80	(1.52–2.13)	322 (20.0)	0.59	(0.49–0.72)
Every day	139 (18.7)	1.36	(1.03–1.81)	480 (64.6)	2.67	(2.10–3.39)	404 (54.4)	2.02	(1.60–2.53)	396 (53.3)	2.14	(1.73–2.65)	127 (17.1)	0.44	(0.34–0.58)
*p* for linear trend		0.0154			<0.0001			<0.0001			<0.0001				

^a^ Adjusted for age, education level, household income, occupation, residence area, current smoking, regular physical activity, year, and usual drinking quantity. ^b^ Adjusted for age, education level, household income, occupation, residence area, current smoking, regular physical activity, year, and drinking frequency. ^c^ Adjusted for age, education level, household income, occupation, residence area, current smoking, regular physical activity, and year. Abbreviations: CI, confidence interval; OR, odds ratio.

**Table 4:** The association between alcohol drinking patterns and components of metabolic syndrome in women.

Variables	Abdominal Obesity			High Blood Pressure			Impaired Fasting Glucose			Hypertriglyceridemia			Low HDL Cholesterol		
	N (%)	OR	95% CI	N (%)	OR	95% CI	N (%)	OR	95% CI	N (%)	OR	95% CI	N (%)	OR	95% CI
Drinking frequency ^a^															
Less than once a month	563 (25.4)	1.00		747 (33.7)	1.00		653 (29.4)	1.00		479 (21.6)	1.00		918 (41.4)	1.00	
Once a month	222 (23.6)	0.98	(0.77–1.25)	280 (29.8)	0.99	(0.77–1.27)	268 (28.5)	1.09	(0.89–1.35)	177 (18.9)	0.83	(0.65–1.05)	326 (34.7)	0.79	(0.66–0.95)
2–4 times a month	348 (18.5)	0.76	(0.62–0.93)	442 (23.5)	0.92	(0.75–1.13)	416 (22.2)	0.96	(0.79–1.17)	302 (16.1)	0.79	(0.64–0.98)	557 (29.7)	0.70	(0.60–0.82)
2–3 times a week	206 (23.5)	0.96	(0.76–1.23)	227 (25.9)	1.11	(0.87–1.42)	221 (25.2)	1.05	(0.82–1.34)	165 (18.8)	0.83	(0.64–1.07)	201 (22.9)	0.46	(0.37–0.58)
More than 4 times a week	72 (29.6)	0.91	(0.62–1.34)	107 (44.0)	2.00	(1.33–3.02)	80 (32.9)	0.93	(0.63–1.37)	61 (25.1)	1.02	(0.65–1.59)	58 (23.9)	0.39	(0.27–0.57)
*p* for linear trend		0.0448			0.0155			0.7631			0.2005			<0.0001	
Usual drinking quantity ^b^															
1–2 drinks	781 (23.5)	1.00		1104 (33.2)	1.00		968 (29.1)	1.00		666 (20.0)	1.00		1277 (38.4)	1.00	
3–4 drinks	304 (21.9)	1.30	(1.06–1.59)	358 (25.8)	1.13	(0.92–1.39)	356 (25.7)	1.12	(0.93–1.35)	243 (17.5)	1.02	(0.82–1.26)	427 (30.8)	0.93	(0.79–1.10)
5–6 drinks	137 (21.0)	1.76	(1.35–2.30)	166 (25.5)	1.89	(1.43–2.50)	141 (21.6)	1.23	(0.96–1.58)	122 (18.7)	1.41	(1.08–1.86)	160 (24.5)	0.87	(0.68–1.11)
7–9 drinks	111 (24.0)	2.02	(1.50–2.73)	122 (26.4)	2.07	(1.51–2.85)	101 (21.9)	1.52	(1.13–2.06)	88 (19.1)	1.21	(0.87–1.68)	117 (25.3)	0.91	(0.70–1.20)
10 or more drinks	78 (23.9)	2.77	(1.87–4.08)	53 (16.3)	1.44	(0.95–2.17)	72 (22.1)	1.70	(1.15–2.53)	65 (19.9)	1.77	(1.17–2.67)	79 (24.2)	0.88	(0.62–1.25)
*p* for linear trend		<0.0001			<0.0001			0.0043			0.0125			0.7857	
Binge drinking frequency ^c^															
None	861 (24.2)	1.00		1219 (34.3)	1.00		1060 (29.8)	1.00		719 (20.2)	1.00		1358 (38.2)	1.00	
Less than once a month	232 (19.1)	1.24	(1.01–1.52)	240 (19.7)	1.12	(0.90–1.39)	248 (20.4)	1.14	(0.93–1.40)	205 (16.9)	1.09	(0.90–1.33)	369 (30.4)	0.84	(0.71–1.00)
Once a month	149 (20.1)	1.58	(1.23–2.02)	158 (21.3)	1.81	(1.38–2.37)	157 (21.2)	1.38	(1.08–1.76)	113 (15.2)	1.09	(0.83–1.44)	177 (23.9)	0.68	(0.54–0.87)
Once a week	121 (23.5)	1.56	(1.15–2.11)	136 (26.4)	1.97	(1.49–2.61)	124 (24.0)	1.31	(0.99–1.72)	109 (21.1)	1.25	(0.93–1.69)	130 (25.2)	0.64	(0.50–0.82)
Every day	48 (36.9)	2.58	(1.63–4.10)	50 (38.5)	2.51	(1.49–4.23)	49 (37.7)	1.97	(1.26–3.09)	38 (29.2)	1.76	(1.08–2.87)	26 (20.0)	0.32	(0.18–0.56)
*p* for linear trend		<0.0001			<0.0001			0.0059			0.2341			<0.0001	

^a^ Adjusted for age, education level, household income, occupation, residence area, current smoking, regular physical activity, menopausal status, year, and usual drinking quantity. ^b^ Adjusted for age, education level, household income, occupation, residence area, current smoking, regular physical activity, menopausal status, year, and drinking frequency. ^c^ Adjusted for age, education level, household income, occupation, residence area, current smoking, regular physical activity, and year. Abbreviations: CI, confidence interval; OR, odds ratio.

## Data Availability

All data files are available from the Korea Centers for Disease Control and Prevention database through the following URLs: https://knhanes.cdc.go.kr/knhanes/sub03/sub03_02_02.do, accessed on 28 December 2021. Anybody, including an international researcher who signs up for membership, can get raw data from the webpage.
